# Wastewater Treatment Using Wood Ash and Cement as Chemical Coagulant

**DOI:** 10.1155/2023/8274687

**Published:** 2023-09-30

**Authors:** Milkessa Ingida, Gurmesa Bedane, Firanbon Adugna, Degefa Nigusu, Mohammed Hussen, Chala Hailu Sime

**Affiliations:** Jimma University, Faculty of Civil and Environmental Engineering, Jimma 378, Ethiopia

## Abstract

Water is essential for daily activities and maintaining human well-being. However, in many less-developed countries, including Ethiopia, the lack of a well-developed wastewater treatment system leads to contaminated surface water. This poses significant risks to human health. To address this problem, wastewater can be treated using locally available materials such as wood ash and cement as chemical coagulants. The objective of this study was to treat wastewater using these materials. The study involved analyzing a 20-liter sample of wastewater from the Awetu River in Jimma City, Ethiopia. The materials used for the treatment included wood ash, cement, and lemon. Various doses of cement and wood ash were prepared and added to the wastewater. The results showed that 5 g was the optimum dosage for effectively treating the wastewater. The treated water at the optimum dosage exhibited significant improvements in turbidity, total dissolved solids, conductivity, and color, meeting drinking water criteria. Overall, the study concludes that locally available materials such as wood ash and cement can be successfully utilized as chemical coagulants for wastewater treatment. This approach offers a viable solution for improving water quality and reducing the risk of waterborne diseases.

## 1. Introduction

Water contamination can occur due to natural environmental factors as well as human activities. Contaminated water can cause health risks to individuals through microbiological, chemical, and physical contamination [1]. Microbiological contamination includes various disease-causing organisms such as bacteria, fungi, and viruses, which can lead to the spread of pathogens and even epidemics, potentially resulting in fatalities [2, 3]. Waterborne diseases refer to infections transmitted through the consumption of contaminated drinking water. While various pathogens can be transmitted through water, bacteria and protozoa are among the most common culprits [4].

Apart from specific substances such as cyanide and nitrate, chemical contamination usually presents a prolonged threat to health. When water quality is compromised in terms of clarity, appearance, or flavor, it might be considered unsatisfactory for consumers. Physical contamination also poses a potential health hazard, as numerous microorganisms are frequently linked with solid particles in water, thus elevating the likelihood of survival and dissemination of microbiological impurities [5].

In order to ensure the safety of water for consumption, all wastewater needs to undergo treatment to remove any potential health risks associated with it [6]. Most treatment systems are designed to address microbiological contamination and physical constituents that hinder acceptability or support microorganism survival, mainly related to suspended solids in the water. Additionally, disinfection is a standard practice included in water treatment processes to further enhance safety [7].

The contamination of drinking water with pathogens presents a significant and widespread health risk to humans globally, leading to numerous disease outbreaks and instances of poisoning resulting from exposure to untreated or inadequately treated water throughout history [8]. Efforts to enhance water quality and sanitation have seen substantial investments from international donors and governments. However, the extension of water supply systems in developing countries has faced challenges, and there are still over 780 million people worldwide lacking access to improved sources of drinking water. Specifically, within this statistic, a significant portion, exceeding 605 million individuals, resides in sub-Saharan Africa, highlighting the region as particularly affected by inadequate access to safe water [9].

As a developing country, Ethiopia has embraced the Millennium Development Declaration, which primarily aims to reduce poverty [10]. Previous studies have highlighted the importance of clean water, proper sanitation, and hygiene in poverty alleviation [11]. The provision of safe drinking water and appropriate treatment methods is a global concern. However, developing countries, including Ethiopia, have been facing insufficient access to safe drinking water from improved sources and inadequate sanitation services [12].

Consequently, individuals continue to rely on unsecured water sources like rivers, streams, springs, and hand-dug wells. Due to their open nature, these sources are highly vulnerable to contamination from floods, birds, animals, and human activities [13, 14]. Moreover, many of these water sources are located near gullies where open defecation is prevalent, leading to the contamination of water by flood-washed waste materials [15]. To ensure environmental safety, a considerable portion of the water utilized by households, industries, and businesses requires treatment before it is discharged back into the environment [16].

The issue of water quality has direct or indirect implications for health. This alarming revelation emphasizes the critical need for global attention and research on water treatment [17]. A substantial portion of the population in developing countries, such as Ethiopia, primarily residing in rural areas, faces significant challenges in accessing safe drinking water and adequate water quality for various purposes, including irrigation. The problem of water quality is pervasive, affecting both urban and rural populations [18].

Access to improved sources of drinking water in Ethiopia is limited, with only 54% of households having access to such sources [19]. Furthermore, a mere 8% of households have improved toilet facilities that are not shared with other households [20]. This issue is prevalent in the Jimma community, resulting in numerous individuals being affected by waterborne diseases like diarrhea and cholera.

Assessing water treatment measures is crucial in determining the availability of improved water sources. The quality of drinking water is deemed acceptable when it meets the requirements in terms of its physical, chemical, and bacteriological parameters, ensuring safety [21]. Chemical coagulants are commonly employed in community drinking water treatment systems, although some applications can be observed in household water treatment methods.

The primary chemicals utilized for coagulation in water treatment include aluminum sulfate (alum), polyaluminium chloride (PAC), alum potash, and iron salts (ferric sulfate or ferric chloride). Additionally, lime (Ca(OH_2_)), lime soda ash (Na_2_CO_3_), and caustic soda (NaOH) are occasionally employed to soften water [22]. Ensuring access to clean drinking water is crucial for human health, national security, and economic prosperity. The coagulation process in water treatment aims to eliminate colloidal particles from wastewater, which can encompass suspended matter and various sizes of solid particles [23].

Ethiopia's current focus, as outlined in the Millennium Development Goal declaration, is poverty alleviation through the effective utilization of water treatment resources [24]. To accomplish this objective, one of the priority areas is the provision of sufficient and high-quality water [18, 25]. Hence, it becomes imperative to find a cost-effective approach for treating contaminated surface water in both rural and urban communities. This research aims to address this issue by utilizing wood ash and cement as chemical coagulants for wastewater treatment.

Obtaining and ensuring a sufficient water supply has always played a critical role in the development of human settlements. In order to make water suitable for drinking and other purposes, various forms of treatment are necessary for all water sources. The main objective of this research aligns with this need and aims to treat wastewater for potable use or drinking and other purposes by employing cement and wood ash. These materials have been chosen due to their effectiveness and easy availability locally. Thus, this study utilizes cement and wood ash as coagulants for wastewater treatment.

## 2. Materials and Methods

The study was conducted in the southwest of the Oromia region, specifically within Jimma City of Ethiopia. The wastewater samples for laboratory analysis were collected from Awetu River, which is situated within Jimma City. The river is located roughly 3 kilometers away from the Jimma Institute of Technology. Its geographic coordinates are approximately 7°40′3″N latitude and 36°50′5″E longitude.

### 2.1. Collection of Raw Material

For this study, a wastewater sample was collected from the Awetu River in Jimma, Ethiopia. The laboratory facilities at Jimma Institute of Technology, specifically the Water Supply and Environmental Engineering Laboratory, were utilized to analyze and measure different properties of the collected wastewater samples. The properties of the water, both before and after the coagulation process, were tested. This allowed for the assessment of the efficiency and effectiveness of the coagulation treatment on the sampled wastewater collected.

### 2.2. Materials and Chemicals Used

In this research, various materials were used to conduct the experiments effectively. These included instruments like a balance for precise measurements, a turbidity meter to assess water clarity, a Crison conductivity meter to measure conductivity, cuvettes for holding samples, a pH meter for acidity testing, beakers for mixing, filter papers for separating particles, specialized jar test equipment, pipettes for accurate liquid transfer, plates for organizing samples, an oven for controlled drying, an incubator for specific environmental conditions, crucibles for heating samples, and various chemicals like lemon for adjustments and treatments. Each of these tools played a crucial role in obtaining accurate data and insights during the study.

### 2.3. Cement and Wood Ash as a Chemical Coagulant

Cement and wood ash, both of which are white fine powders, were employed in the treatment of wastewater in this study. Ash can come in various forms, including wood ash, volcanic ash, coal ash (fly ash), cremation ash, and seaweed ash. For this research, wood ash was specifically used after undergoing a sieving process. In addition, wood ash can be used as a chemical coagulant. It contains various minerals, including alumina (Al_2_O_3_), up to 15 percent iron oxide (Fe_2_O_3_), and ideally no more than about 6 percent silica (SiO_2_). The primary compound in wood ash responsible for its coagulating properties is calcium aluminate (CaO·Al_2_O_3_). The primary compound responsible for cement's binding properties is calcium aluminate (CaO. Al_2_O_3_) [26].

Wood ash, on the other hand, contains several compounds such as calcite (CaCO_3_), lime (CaO), and calcium chlorate hydrate (Ca(ClO)_2_.3H_2_O) [27]. The primary compound found in wood ash is calcium carbonate or calcite. When cement and wood ash are used together in water treatment processes, their combined properties and chemical compositions can contribute to the coagulation of particles and the removal of impurities from wastewater. The specific characteristics of cement and wood ash make them effective materials for treating contaminated water and improving its quality.

Wood ash and cement are derived from different sources and production processes. Wood ash is obtained through the burning of wood, while cement is produced in industrial settings and comprises lime, silica, alumina, and iron oxide. The particle size of wood ash may vary depending on the source, with some ashes being finer or coarser than cement particles. When a mixture of cement and wood ash was added to high turbidity water or wastewater, they settled at the bottom of the water, allowing for the purification of water, with the purified water being collected from the top.

When only wood ash was used as a sole coagulant, it tended to form gel-like lumps when mixed with wastewater, which impeded the treatment process. To address this issue and minimize the formation of gel-like lumps, a mixture of both cement and wood ash was utilized as a chemical coagulant. The addition of cement helped to prevent the formation of gel-like lumps. Moreover, using cement alone as a coagulant would be more expensive compared to wood ash.

### 2.4. Preparation of Cement and Wood Ash as a Coagulant

In this study, the powder of cement and wood ash was prepared separately. The fine powder of wood ash was obtained by using a mortar and pestle to grind the ash into a finely powdered form. This wood ash powder was then directly mixed with cement, which acted as a coagulant for wastewater treatment purposes. The ratio of cement to wood ash can vary depending on specific treatment goals, characteristics of the wastewater, and local conditions. Generally, the mixture may consist of cement and wood ash in proportions ranging from 1 : 1 to 1 : 3 by weight. However, it is crucial to note that precise ratios should be determined through laboratory testing and empirical observation to ensure optimal performance in a given wastewater treatment application. For this study, cement and wood ash of 1 : 3 by weight were used as a coagulant in the wastewater treatment process. When cement and wood ash are mixed and added to wastewater, it forms a weak base and part of salt is settled down. The prepared cement and wood ash powder mixture was added to this fixed volume of wastewater for coagulation. This allowed for the assessment of the effectiveness of the coagulation process in treating the wastewater.

**Figure 1:**
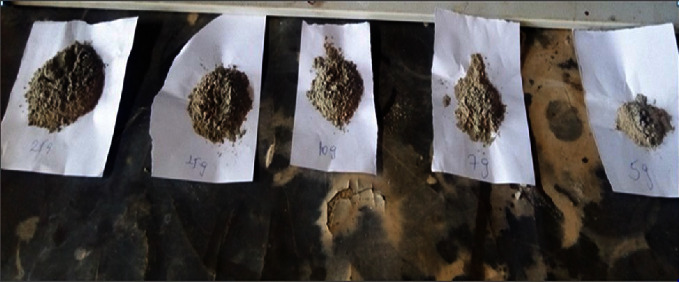
Dosage of wood ash and cement.

To determine the appropriate dose for wastewater treatment, various amounts of cement and wood ash (5, 7, 10, 15, and 25 grams) were measured using a balance and mixed with the sample wastewater. The mixture was then placed on a shaker and agitated for several minutes. This ensured thorough mixing and interaction between the coagulant and the wastewater.  Figure 1 shows dosage of wood ash and cement.

### 2.5. Coagulation Experiments

In this study, a total of 20 liters of wastewater samples were collected from the Awetu River for coagulation-flocculation analysis. To conduct the analysis, the collected water sample was dispensed into five separate beakers, with each beaker containing 500 ml of the sample water. Dividing this sample into beakers of the same volume is a deliberate choice to establish controlled experimental units. This fixed volume not only facilitates precise dosage measurements but also allows for consistent treatment across samples. Such careful handling of the sample ensures that any observed variations in the coagulation process are attributable to the dosage levels and not influenced by sample size.

**Figure 2:**
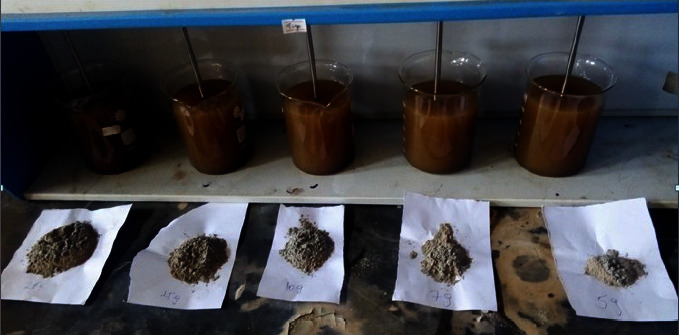
Jar test and dosage.

The coagulation-flocculation process was carried out using a jar test apparatus. The measured dosage of cement and ash (5 g, 7 g, 10 g, 15 g, and 25 g) was added to the five individual beakers, each containing 500 ml of the water sample. This setup enabled the evaluation of the effectiveness of different coagulant dosages in achieving coagulation-flocculation in wastewater treatment.  Figure 2 shows the jar test and dosage.

Once the desired amount of coagulant mixture was added to the turbid water sample, the blades of the jar test apparatus were adjusted based on the intended mixing speed for both the coagulation and flocculation tests.

For the coagulation test, which involves rapid mixing, the blades were set to rotate at a speed of 260 rotations per minute (rpm). This speed was maintained for 5 minutes to ensure thorough mixing of the added coagulant with the colloidal particles present in wastewater. This rapid mixing promotes the destabilization of the particles and initiates the coagulation process.

Following the coagulation process, the next step involved flocculation, which requires slower mixing. To allow the destabilized particles to agglomerate and form larger flocs that can settle more rapidly, the rotation speed of the blades was reduced. By decreasing the rotation speed of the stirrers, the particles were encouraged to agglomerate and form flocs, facilitating their settling. In this case, stirrers were allowed to rotate at a speed of 90 rpm for 15 minutes.

Following the flocculation process, the samples were allowed to settle for 30 minutes. During this settling period, the particles settled at the bottom of the container, allowing the clear supernatant to form on top. After the settling processes were completed, samples of the clear water were collected for the analysis of their turbidity, pH, conductivity, color, and total dissolved solids (TDS).

#### 2.5.1. Water Parameter Conducted

pH tests were conducted to assess the effect of the coagulation process on the water pH level. Each coagulant has an optimal pH range at which it works best, typically between 6.5 and 8.5. Lower pH levels tend to favor organic removal, while higher pH levels promote inorganic removal. pH levels were monitored and controlled by adjusting the coagulant dosage levels. The pH of the water samples is measured using a calibrated Crison pH meter, ensuring accurate pH readings for analysis.

The turbidity test is a method used to quantify the presence of suspended matter in a water sample, which can include both organic and inorganic substances. Turbidity serves as an important indicator of contamination levels in water, making it crucial to minimize turbidity throughout the treatment process. To conduct the turbidity test, a sample of turbid water is poured into a 25 ml cuvette and inserted into a turbidity meter. The turbidity meter measures the intensity of light scattered by the suspended particles in the water sample. The resulting turbidity value is displayed on the instrument's LCD panel and is typically expressed in Nephelometric Turbidity Units (NTU).

By evaluating the turbidity levels before and after treatment, the efficiency of the coagulant dosage can be determined, allowing for adjustments to be made as needed to achieve the desired reduction in turbidity. Minimizing turbidity is crucial to ensure water quality and remove potential contaminants. The percentage of turbidity removal is given by

(1) $$\begin{array}{c} \%\text{Turbidity removal}=\frac{\text{Initial turbidity}-\text{Final turbidity}}{\text{Initial turbidity}}\times 100\%\text{.} \end{array}$$

A conductivity test is conducted to measure the total dissolved solids (TDS) in water. This test helps determine the presence of both cations and anions in the water sample before and after treatment. To perform the conductivity test, a conductivity meter is used. The water sample is poured into a beaker, ensuring that the meter probe does not touch the sides or bottom of the beaker. The meter is then carefully inserted into the water, allowing it to stabilize. The reading of conductivity is displayed on the LCD panel of the meter once it has reached equilibrium. Conductivity tests provide valuable information about the level of dissolved ions, salts, and other substances in the water.

By comparing the conductivity measurements before and after treatment, the effectiveness of the treatment process in reducing the presence of dissolved solids can be determined. To ensure accurate conductivity measurements, it is important to use a properly calibrated Crison Conduct meter. Regular calibration of the meter helps ensure reliable and precise readings, allowing for accurate monitoring of the water's conductivity levels throughout the treatment process.

Total dissolved solids (TDS) refer to all the solid substances that are dissolved in water. In potable water, TDS mainly consist of inorganic salts, minute amounts of organic matter, and dissolved gases. The presence of high TDS levels in water can be reduced through processes like oxidation, settling, and filtration and can also be completely removed through distillation. TS refer to total dissolved solids and suspended solids in raw water. The calculation result shows the TS of water before and after adding coagulants. The percentage turbidity removal for the added different dosage of CWA coagulant was determined from the relation

(2) $$\begin{array}{c} \text{TS}=\frac{\left(W2-W1\right)}{\text{Vt}}\text{,} \end{array}$$

where TS = total dissolved solids in mg/l, *W*1 = mass of crucible in grams, *W*2 = mass of crucible with sample water after oven-dried in grams, and *V*_*t*_ = total volume of sample water in liter (*l*).

Color, taste, and odor are important characteristics to consider when assessing water quality. The color of wastewater collected from the Awetu River, for example, is determined using a spectrometer. Prior to using the spectrometer, calibration is performed using distilled water as a reference. The wastewater sample is then placed in the spectrometer using a sample set, and the peak absorbance of the sample water is read from the graph displayed on the LCD spectrum. Taste and odor are subjective assessments, referring to any taste or smell in water that deviates from what is considered acceptable by the consumer.

#### 2.5.2. The Sample Wastewater Parameters

The measurements of various water quality parameters provide valuable insights into the condition of the water being examined. The collected wastewater has a turbidity of 145 NTU. A turbidity level of 145 indicates that the water may contain suspended particles or impurities. It has a salinity of 151.8 mg/l, which indicates a relatively high level. It has a resistivity of 0.32 kῼ; a low resistivity signifies that the water is not very resistant to electrical flow. The dissolved oxygen level of 2.17 mg/l present in the sample water may be a concern for aquatic organisms that rely on higher oxygen concentrations. An absorbance value of 0.863 suggests the presence of substances that can absorb light. With a transitivity of 13.7, the water allows a moderate amount of light to pass through. The measurement of total solids at 6.4 indicates the presence of solid particles in the water. These parameters collectively offer a comprehensive assessment of the water quality, highlighting its suitability for various purposes and its potential impact on the environment.  Table 1 shows the parameters of sample wastewater before the addition of coagulant.

## 3. Results and Discussion

### 3.1. Cement and Wood Ash in Turbidity Removal of Wastewater

**Figure 3:**
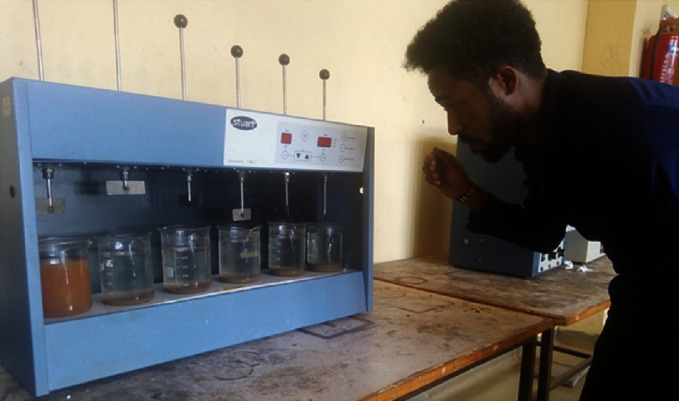
Experimental result after coagulation and flocculation process.

Experimental results after coagulation and flocculation processes with different dosages are shown in Figure 3. During the jar test experiment, different coagulant dosages were added to a 500 ml of sample water with an initial turbidity of 145 NTU. After undergoing the coagulation-flocculation and clarification processes, the supernatant sample water was collected for turbidity analysis. The results showed that the addition of the coagulant dosage led to a reduction in turbidity. The turbidity values after treatment were measured as follows: 5.22 NTU for the 5 g dosage, 7.98 NTU for the 7 g dosage, 13.63 NTU for the 10 g dosage, 22.00 NTU for the 15 g dosage, and 27.84 NTU for the 25 g dosage. As shown in Figure 3, it is clear that the turbidity of the wastewater is significantly decreased.

The experiment has shown that the turbidity of the raw water was reduced to 5.22 NTU with a removal efficiency of 96.4% using a 5 g dosage of cement and wood ash. At this dosage, cement and wood ash are active coagulants. Therefore, 5 g is the optimum dosage for turbidity removal. This indicated that cement and wood ash can be used as chemical coagulants in turbidity removal.

Similarly, as the concentration of the coagulant increased, we see a gradual decrease in removal efficiency, indicating that higher concentrations do not necessarily yield better results. Even at the highest concentration of 25 g, the removal efficiency was 80.8% as indicated in Table 2.

### 3.2. Cement and Wood Ash Coagulant on the pH

The experiment involved testing the pH levels of treated water before and after the addition of lemon, along with varying dosages of coagulants, as shown in Table 3. The initial measured pH value of sample wastewater was 7.91 at room temperature. After adding coagulants, the pH levels increased for each dosage: 5 g resulted in 8.7, 7 g in 8.92, 10 g in 9.16, 15 g in 9.32, and 25 g in 9.67. These values slightly exceeded the recommended pH range of 6.5 to 7.5 according to WHO standards [28]. So, it cannot satisfy the recommended values. To address this, an acid solution was introduced to the water samples to neutralize them. For instance, 5 ml of lemon was added to 500 ml of water treated with 5 g of coagulant, resulting in a pH of 6.97. This adjustment brought the water within the acceptable pH range for domestic use, demonstrating the effectiveness of the treatment process.

### 3.3. Cement and Wood Ash Coagulant on Conductivity

The ability of water to conduct electricity is determined by its conductivity, which is influenced by the presence of positively charged ions (cations) and negatively charged ions (anions) in the water. When water contains high levels of ions, it typically exhibits lower electric conductivity. Before the coagulants were added to the turbid water, the conductivity value of the water sample was measured as 580 *μ*s/cm. After the addition of the coagulants with varying dosages per 500 ml of water as 5 g, 7 g, 10 g, 15 g, and 25 g, the resulting conductivity values of the treated water are 265, 300, 381, 437, and 517 *μ*s/cm, respectively. A lower value of conductivity is the property of clean water. The values of the conductivity of treated water with different dosages show the feasible results to be required for water quality as per WHO standards which are in the range of 200 *μ*s/cm to 800 *μ*s/cm [28]. The conductivity of the treated water sample is shown in Table 4.

### 3.4. Cement and Wood Ash Coagulant on Color Removal

The experiment involved treating 500 ml of turbid water with an initial turbidity of 145 NTU with a light absorbance value of 0.863. The absorbance of light was measured using an electrophotometer, and the results displayed varying degrees of absorbance. As shown in Table 5, when 5 g of coagulant was used, the absorbance was only 0.007, resulting in an impressive color removal of 99.18%. Similarly, with 7 g of coagulant, the absorbance was 0.009, indicating a color removal of 98.95%. As the dosage increased, the absorbance values also rose, but the percentage of color removal slightly decreased. For instance, at 25 g of coagulant, the absorbance was 0.097, and the color removal percentage was 88.76%. It can be observed that the absorbance values increased as the dosage of the coagulant increased. This indicates that the percentage of color removed from the water decreased with higher coagulant dosages. In other words, higher doses of the CWA coagulant were more effective in removing unnecessary color from the raw water.

This finding demonstrates that the CWA coagulant is particularly effective in addressing and reducing color-related issues in wastewater, providing valuable information for optimizing the treatment process for improved water quality.

### 3.5. Cement and Wood Ash Coagulant on the Removal of Dissolved Oxygen

Dissolved oxygen (DO) is used to describe the amount of oxygen dissolved in a unit volume of water. It is essential for the maintenance of healthy lakes and rivers. In healthy water bodies such as lakes and rivers or streams, the dissolved oxygen is about 10 ppm. The minimum level of 3 to 5 mg/l is desirable for the survival of aquatic life. In the experiment of wastewater, the DO value before treatment was 2.17 mg/l. After treatment, as the dosage of coagulant increased, the DO value also increased, and at 5 g of dosage, the DO value after treatment was 3.26 mg/l. The test results show that the treated water is in a healthy condition and suitable for aquatic life. After the addition of coagulant to wastewater, the dissolved oxygen of the sample water was discussed as listed in Table 6.

### 3.6. Cement and Wood Ash Coagulant on the Salt

Salinity refers to the measurement of dissolved salt content in water. Seawater is known to have a salinity of approximately 3500 mg/l, while freshwater typically has a salinity of about 1000 mg/l [28]. In the experiment conducted, prior to the addition of the coagulant, the initial salt concentration in the water was measured to be 151.8 mg/l at room temperature. After the coagulant was added, the salt concentrations varied depending on the dosage used. Specifically, the salt concentrations were measured as 897 mg/l, 960 mg/l, 1186 mg/l, 1600 mg/l, and 2203 mg/l for different doses of the coagulant in 500 ml of the sample water.

**Figure 4:**
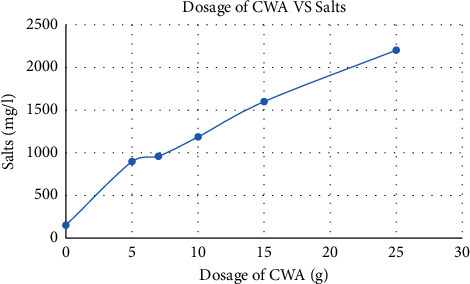
Effect of CWA on salt in water treatment.

These results indicate that the addition of the coagulant had an impact on the salt concentration in the water. The varying doses of the coagulant resulted in different levels of salt in the treated water sample. It is important to note that the specific coagulant used and the dosages applied may have contributed to these variations in salt concentration. From Figure 4, as the amount of the dosage increased, the salinity of the water also increased. When the salinity is less than 250 mg/l, it causes diseases like cardiovascular disease, heart disease, and kidney disease, and when it is above 1000 mg/l, it also causes diseases. So, for the sample of 5 g dosage with 500 ml of raw water, the obtained value was 897 mg/l and it satisfies the WHO ranges.

### 3.7. Cement and Wood Ash Coagulant on the Resistivity of the Sample Water

**Figure 5:**
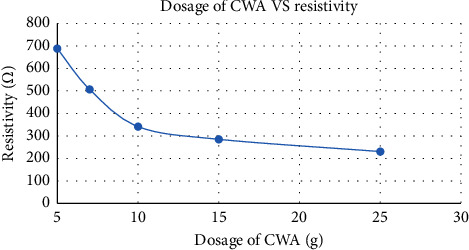
The resistivity of treated water with different dosages of coagulant.

Resistivity in water is the measure of the ability of water to resist an electrical current, which is directly related to the number of dissolved salts in the water. Water with a high concentration of dissolved salts will have a low resistivity. The appropriate resistivity of clean water is recommended as 500 Ω–1500 Ω. As shown in Figure 5, it is noteworthy that initially, the water exhibited a low resistivity of 0.32 kΩ across all coagulant dosages. However, after the coagulation process, there was a significant increase in resistivity values. For instance, with a 5 g coagulant dosage, the resistivity surged to 688 Ω. Similarly, as the dosage increased, the final resistivity values followed suit, showcasing a trend of improved resistance to electrical flow in the treated water. The results suggest that the use of cement and wood ash coagulant can significantly contribute to achieving the desired resistivity levels for quality water treatment.

### 3.8. Cement and Wood Ash Coagulant on the Transitivity

Transitivity is the ability of water to transmit light. A high percentage of transparency indicates more transmitted light.  Table 7 illustrates the initial and final transitivity percentages for various coagulant dosages. Initially, the water exhibited a transitivity of 13.7%, indicating a moderate ability to transmit light. Following the coagulation process, there was a notable increase in transitivity percentages across all dosages. For example, with a 5 g coagulant dosage, the final transitivity reached an impressive 98.3%. Similarly, as the dosage increased, the final transitivity percentages remained notably high. This demonstrates the effectiveness of the coagulation process in enhancing the water's ability to transmit light, which is crucial for maintaining water clarity. The results suggest that the use of cement and wood ash coagulant can significantly contribute to achieving the desired transitivity levels for quality water treatment.

### 3.9. Experimentation of Cement and Wood Ash Coagulant in Reducing TDS

The TDS values of sample water after the addition of coagulant are presented in Table 8. TDS refer to the materials that are completely dissolved in water; this solid is filterable, and it is the residue after evaporation of the filterable sample. As indicated in Table 8, as the coagulant dosage increased, there was a notable rise in TDS concentrations. For instance, at a 25 g coagulant dosage, the TDS reached 3767 mg/l. This indicates that higher coagulant dosages led to an increase in the concentration of dissolved solids in the treated water. It is important to note that while the coagulation process effectively reduced turbidity, it also resulted in an elevation of TDS levels. The amount of TDS for freshwater is <1500 mg/l. The TDS value is between 1500 mg/l to 5000 mg/l in brackish water and >5000 mg/l in saline water [28]. At a dosage of 5 g of CWA coagulant in 500 ml, the obtained TDS value was 987 mg/l. This demonstrates that CWA is effective in removing TDS from raw water.

### 3.10. Total Solids (TS) of Wood Ash and Cement

Another significant characteristic of clarified (treated) water is its reduced total solids (TS). After applying the coagulant to clear the initially turbid water with a turbidity of 145 NTU, the TS level was 6.4 mg/l. However, at the ideal dose of 5 grams per 500 ml of raw water, the TS value notably dropped to 0.88 mg/l. This demonstrates the coagulant's effectiveness in reducing the total solids content in the treated water, underlining its role in enhancing water quality.  Table 9 indicates the TS of wastewater before adding CWA.

The TS value of wastewater before adding coagulant and after oven-dried for 1 hr at 105°C was 6.4 mg/l. After using various amounts of CWA coagulant on a 500 ml sample of wastewater, the sample was dried in an oven at 105°C for 1 hour. The results, shown in Table 10, indicate that as the dosage of CWA increased, the total solids (TS) content also increased. At 5 grams per 500 ml of raw water, the TS concentration was 0.88 mg/l. This indicates that the CWA coagulant effectively reduced the total solids content in the treated water, which is crucial for enhancing water quality and suitability for various purposes. The trend in the data underscores the potential of CWA as an effective coagulant in wastewater treatment.

### 3.11. Recommended Value with Obtained Value at 5 g Dosage of CWA

Based on the experimental results obtained at the optimum dosage, all the parameters measured were found to be within the standard range for drinking water quality. The comparison between the recommended values and the obtained values is presented in Table 11.

Based on these results, it can be concluded that the treatment process, particularly at the optimum dosage, successfully met the recommended values for drinking water. The turbidity, pH, conductivity, dissolved oxygen, absorbance, transmittance, TDS, resistivity, and salt content were all within acceptable limits for safe drinking water quality. This indicates the effectiveness of the treatment in achieving suitable water quality standards.

## 4. Conclusions

This study was conducted in Jimma City of Ethiopia, specifically focusing on wastewater samples collected from the Awetu River, situated approximately 3 kilometers away from the Jimma Institute of Technology. The study aimed to evaluate the effectiveness of cement and wood ash as chemical coagulants in treating wastewater. Through a series of experiments, various properties of the water before and after treatment were analyzed. The results showed that the addition of the coagulant led to a reduction in turbidity, indicating improved water clarity. Additionally, the treatment process had positive effects on pH levels, conductivity, dissolved oxygen, color removal, and total dissolved solids (TDS), all of which are crucial factors in assessing water quality. Notably, at an optimal dosage of 5 grams per 500 milliliters of raw water, the total solids content was significantly reduced, demonstrating the coagulant's effectiveness in purifying the water. Overall, this study provides valuable insights into the potential use of cement and wood ash as coagulants for wastewater treatment, showing promising results in achieving safe and suitable water quality standards.

## Figures and Tables

**Table 1:** Parameters of sample wastewater before the addition of coagulant.

Test	Measurement	Unit
Turbidity	145	NTU
pH	7.91	pH meter
TDS	1161.5	mg/l
Conductivity	580	*μ*s/cm
Salt	151.8	mg/l
Resistivity	0.32	kῼ
Dissolved oxygen	2.17	mg/l
Absorbance	0.863	—
Transitivity	13.7	—
Total solid	6.4	mg/l

**Table 2:** Percentage of turbidity removal with different dosages of CWA.

CWA coagulant concentration (g/500 ml)	Initial turbidity (NTU)	Final turbidity (NTU)	Percentage of turbidity removal
5	145	5.22	96.4
7	145	7.98	94.8
10	145	13.63	90.6
15	145	22.00	86.8
25	145	27.84	80.8

**Table 3:** pH of the treated water before and after addition of lemon.

Treated water sample (g/500 ml)	pH value	Efficiency of the lemon on the pH
5	8.7	6.97
7	8.92	7.23
10	9.16	7.45
20	9.32	7.62
25	9.67	7.95

**Table 4:** The conductivity of the treated water with different coagulant dosages.

Treated water sample (g/500 ml)	Conductivity value (*μ*s/cm)
5	265
7	300
10	381
15	437
25	517

**Table 5:** Absorbance and percentage color removal of different coagulant dosages added.

No.	Sample	Coagulant added (g/500 ml)	Absorbance	% color removal
1	S1	5	0.007	99.18
2	S2	7	0.009	98.95
3	S3	10	0.073	91.53
4	S4	15	0.081	90.6
5	S5	25	0.097	88.76

**Table 6:** Value of dissolved oxygen after treated with different dosages of CWA.

No.	Sample of coagulant (g/500 ml)	Initial dissolved oxygen (mg/l)	Final dissolved oxygen (mg/l)
1	5	2.17	3.26
2	7	2.17	3.95
3	10	2.17	5.25
4	15	2.17	5.46
5	25	2.17	5.52

**Table 7:** Transitivity of treated water with different dosages of CWA.

No.	Sample of coagulant (g/500 ml)	Initial transitivity (%)	Final transitivity (%)
1	5	13.7	98.3
2	7	13.7	97.9
3	10	13.7	84.6
4	15	13.7	82.9
5	25	13.7	80.0

**Table 8:** TDS of treated water after adding coagulant.

No.	Sample	Coagulants added (g/500 ml)	TDS (mg/l)
1	B1	5	987
2	B2	7	1126
3	B3	10	2065
4	B4	15	2439
5	B5	25	3767

**Table 9:** TS of wastewater before adding CWA.

Sample of water before treatment	Volume of sample (ml), *V*_*t*_	Mass of crucible (g), *W*_1_	Mass of crucible with 25 ml of sample water after oven-dried (g), *W*_2_	TS (mg/l)
S-0	25	56.069	56.229	6.4

**Table 10:** TS of treated wastewater after adding CWA with different dosages.

Sample water after treated	Dosage of CWA (g)	The volume of sample water (ml), *V*_*T*_	Mass of crucible (g), *W*_1_	Mass of crucible with 25 ml of sample water after oven-dried (g), *W*_2_	TS (mg/l)
S-1	5	25	50.426	50.448	0.88
S-2	7	25	52.644	52.684	1.60
S-3	10	25	52.997	53.054	2.28
S-4	15	25	49.513	49.598	3.40
S-5	25	25	49.523	49.639	4.64

**Table 11:** Comparison of recommended values with obtained values.

Parameters	Recommended value	Obtained value	Unit
Turbidity	5 to 15	5.22	NTU
pH	6.5 to 7.5	6.9	—
Conductivity	200 to 800	265	*μ*s/cm
Dissolved oxygen	3 to 5	3.26	mg/l
Absorbance	100%	99.18%	—
Transitivity	100%	98.3%	—
TDS	<1500	987	mg/l
Resistivity	500 to 1500	688	Ω
Salt	<1000 m	897	mg/l

## Data Availability

All the data are included in the article, and if needed, they will be submitted upon request.
